# Integrating multi-dimensional data to reveal the mechanisms and molecular targets of baikening granules for treatment of pediatric influenza

**DOI:** 10.3389/fmolb.2025.1637980

**Published:** 2025-07-11

**Authors:** Zhaoyuan Gong, Qianzi Che, Mingzhi Hu, Tian Song, Lin Chen, Haili Zhang, Ning Liang, Huizhen Li, Guozhen Zhao, Lijiao Yan, Xuefei Zhang, Bin Liu, Jing Guo, Nannan Shi

**Affiliations:** Institute of Basic Research in Clinical Medicine, China Academy of Chinese Medical Sciences, Beijing, China

**Keywords:** pediatric influenza, baikening granules, bioinformatics, machine learning, network pharmacology, molecular docking, molecular dynamics simulation

## Abstract

**Background:**

Children are the main group affected by the influenza virus, posing challenges to their health. The high risk of viral variability, drug resistance, and drug development leads to a scarcity of therapeutic drugs. Baikening (BKN) granules are a marketed traditional Chinese medicine used to treat children’s lung heat, asthma, whooping cough, etc. Therefore, exploring the potential mechanisms of BKN in treating pediatric influenza is of great significance for discovering new drugs.

**Methods:**

Through the database, we obtained differentially expressed genes (DEGs) between pediatric influenza and healthy samples, identified the components of BKN, and collected the targets. Target networks were built with the purpose of screening both targets and key components. Pathway and function enrichment were conducted on the relevant targets of BKN for treating pediatric influenza. BKN-related hub genes for influenza were discovered through DEGs, weighted gene co-expression network analysis (WGCNA), BKN-cluster WGCNA, and machine learning model. The accuracy of prediction efficiency and the value of BKN-related hub gene were validated through analysis of external datasets and receiver operating characteristics. Ultimately, simulations using molecular docking and molecular dynamics were used to forecast how active components will bind to hub genes.

**Result:**

A total of 20 candidate active compounds, 58 potential targets, and 3,819 DEGs were identified. The target network screened the top 10 key components and 6 core targets (PPARG, MMP2, GSK3B, PARP1, CCNA2, and IGF1). Potential target enrichment analysis indicated that BKN may be involved in AMPK signaling pathway, PI3K Akt signaling pathway, etc., to combat pediatric influenza. Subsequently, two hub genes (OTOF, IFI27) were obtained through WGCNA, BKN-cluster WGCNA, and machine learning models as potential biomarkers for BKN-related pediatric influenza. Two hub genes were found to have primary diagnostic value based on ROC curve analysis. Molecular docking confirmed the binding between BKN and hub gene. Molecular dynamics further revealed the stable binding between Peimisine and hub genes.

**Conclusion:**

BKN may alleviate pediatric influenza via key components targeting core targets (PPARG, MMP2, GSK3B, PARP1, CCNA2, and IGF1) and hub genes (OTOF, IFI27), with the involvement of feature genes-related pathways. These results have potential consequences for future research and clinical practice.

## 1 Introduction

Influenza, caused by the influenza virus (classified under the Orthomyxoviridae family (Xie et al., 2025)), is a respiratory infection that annually results in 3–5 million severe cases and leads to 290,000 to 650,000 deaths worldwide (Troeger et al., 2019). Every year, tens of thousands of children under the age of 5 die from influenza-related respiratory diseases worldwide (Iuliano et al., 2018). Influenza A and B serve as the primary culprits behind respiratory disease outbreaks, frequently resulting in heightened rates of hospitalization and mortality (Suárez-Sánchez et al., 2025). Influenza viruses undergo frequent antigenic drift and antigenic variation, allowing them to evade pre-existing herd immunity and facilitate rapid transmission, thereby causing seasonal influenza outbreaks and global influenza pandemics (El Guerche-Séblain et al., 2019). Children, owing to their compromised immune system and suboptimal vaccination rates, exhibit heightened susceptibility to influenza and are at an elevated risk of developing severe manifestations (Gounder and Boon, 2019). During the annual influenza season, the prevalence of flu among children typically ranges from 20% to 30%. However, in certain seasons with high incidence rates, the annual infection rate of flu in children can escalate to approximately 50% (Glatman-Freedman et al., 2012). The burden of pediatric influenza has a profound impact on individuals and society (Wodniak et al., 2025), including direct medical costs and indirect costs resulting from parents potentially contracting influenza from their children.

In China, traditional Chinese medicine (TCM) preparations also played a core role against influenza (Yang et al., 2025). Bai-Ke-Ning (BKN) granule is a Chinese medicine which had been on the market, and was used to treat *pertussis* in children. BKN has the effect of clearing heat, eliminating phlegm, relieving cough, and relieving asthma. This prescription consists of 3 various kinds of TCMs, such as *Fritillaria ussuriensis Maxim.* (Ping-Bei-Mu), *Baphicacanthus cusia(Nees)Bremek.* (Qing-Dai), and *Ginkgo biloba L.* (Bai-Guo-Ren). Qing-Dai has the function of purging fire, clearing heat, detoxifying, analgesic, and anti-inflammatory (Zeng et al., 2022). Bai-Guo-Ren has the effects on anti-inflammatory and antioxidant. Alkaloids in Ping-Bei-Mu are the main bioactive ingredients, which have the effects of expectorating, relieving asthma and relieving cough (Wang et al., 2018). In TCM, the occurrence of influenza is believed to be linked to pestilence infections. TCM has the characteristics of multiple components and multiple targets, which reflects the thought of “treating with one formula for multiple diseases” in TCM. Therefore, based on its efficacy and modern pharmacological research, BKN may be a potential therapeutic drug for pediatric influenza.

Network pharmacology can explain the pharmacological mechanism from the perspective of multiple targets and multiple ways, explore the target and pathway relationships between drugs and diseases, and clarify the complex mechanism of TCM on this basis (Berger and Iyengar, 2009; Vithalkar et al., 2025). In recent years, weighted gene co-expression network analysis (WGCNA) has often been used in various omics studies to screen hub genes closely related to diseases (Zhao et al., 2021; Zhou et al., 2023). The advantages of WGCNA include (i) mining biological functional modules rather than individual genes. (ii) In combination with clinical phenotypes, identify the key modules. (iii) Identifying the hub gene is of greater biological significance and potential application value. Machine learning techniques are often combined with bioinformatics methods, as well as databases and biological networks, to enhance training and validation, identify the best interpretable features, and enable feature and model investigations (Mirza et al., 2019; Auslander et al., 2021). Among them, random forests (RF), the least absolute shrinkage and selection operator (LASSO), and support vector machine-recursive feature elimination (SVM-RFE) is the most used. Meanwhile, the comprehensive use of multiple machine learning methods can significantly improve the prediction accuracy and model robustness. Sensitivity analysis aims to systematically assess the extent and robustness of the impact of these choices and data uncertainties on the final research results. The receiver operating characteristic (ROC) curve is used to evaluate the overall diagnostic performance of the test and compare the performance of two or more diagnostic tests (Nahm, 2022). The reasons for conducting sensitivity analysis in bioinformatics research include (i) evaluating the robustness of the results. (ii) Identifying the dependency of key parameters/methods. (iii) Addressing data quality and noise issues. (iv) Verifying the newly developed algorithm/process. (v) Enhancing the transparency and reproducibility of research. Molecular docking involves the use of computers to design drugs, commonly utilized for predicting the interactions and binding affinity between proteins and ligands (Kitchen et al., 2004). Furthermore, molecular dynamics simulations (MD) can be utilized to investigate the interactions between proteins and ligands, as well as the fluctuations in residue positions during protein movement (Grewal et al., 2025; Zhang et al., 2025). This approach provides valuable insights into the dynamic processes involved in protein function (Xue et al., 2016). Hence, in this research, we employed poly-bioinformatics to anticipate distinct therapeutic targets and signaling pathways for BKN while exploring potential mechanisms associated with influenza.

**FIGURE 1:**
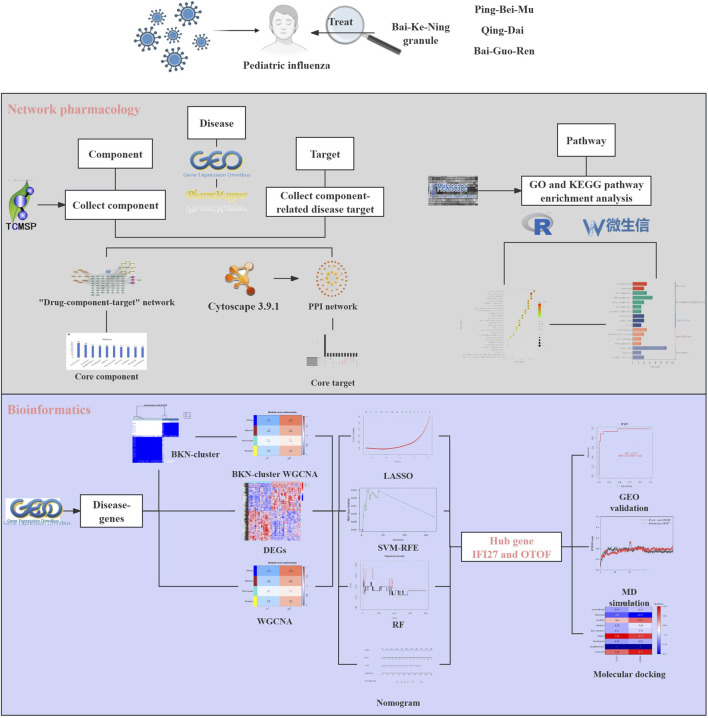
Flow diagram of this study.

This study aims to investigate the mechanism of BKN granule in the treatment of pediatric influenza through bioinformatic, machine learning, network pharmacology, molecular docking technology, and MD. The findings will serve as theoretical references for future experimental research and clinical applications. The steps involved in this investigation are illustrated in Figure 1.

## 2 Materials and methods

### 2.1 Collection of active ingredients and targets in BKN

To gather information on the active ingredients of BKN, we conducted a search using keywords such as “Qingdai,” “Pingbeimu,” and “Baiguoren.” This search was performed on the TCM system pharmacology technology platform (TCMSP, http://tcmspw.com/tcmsp.php) and the China national knowledge infrastructure (CNKI) reviews (Ru et al., 2014). To filter out irrelevant components, we applied specific criteria based on oral bioavailability (OB) ≥ 30% and drug-likeness (DL) ≥ 0.18. Once the active components were identified through screening in the TCMSP database, their SDF structure formats were obtained from the PubChem database (https://pubchem.ncbi.nlm.nih.gov/). We used PharmMapper database (http://www.lilab-ecust.cn/pharmmapper/) (Liu et al., 2010) to predict the potential targets of BKN through SDF structure. Further analysis was carried out by selecting BKN component targets with a Norm Fit value of ≥0.5. For standardization purposes, we selected species “*Homo sapiens*” in uniport (https://www.uniprot.org/) (Uszkoreit et al., 2021) to convert the Uniprot ID into the gene symbol.

### 2.2 Collection and procession of differentially expressed genes in influenza

We obtained microarray data for influenza from two sources, GSE34205 and GSE42026, which were downloaded from the GEO database. The microarray platforms used were GPL6947 (Illumina HumanHT-12 V3.0 expression beadchip) and GPL570 (Affymetrix Human Gene Expression Array). These datasets included samples from pediatric influenza patients as well as healthy individuals without any other diseases. Both groups had a sample size of more than 10. We obtained gene expression and clinical data, and performed gene symbol annotation and data correction using Perl code. GSE34205 (Ioannidis et al., 2012) was used as the analysis set. This study excluded children suspected or confirmed to have multiple bacterial infections, potential chronic diseases (i.e., congenital heart disease or renal insufficiency), immunodeficiency, or receiving systemic steroids or other immunomodulatory therapies. Among them, all influenza virus infected individuals were confirmed and diagnosed through microbiology, and all blood samples were collected within 42–72 h after hospitalization. A total of 28 influenza virus patients were included in the study, with a median time of 5.5 (1.4–21) months. Blood samples from 12 healthy children with a median age of 18.5 (10.5–26) months who underwent elective surgery or outpatient visits were used as control samples (Supplementary Table S1). And GSE42026 (Herberg et al., 2013) was used as the validation set, which includes 19 H1N1/09 samples and 33 healthy controls. The LIMMA software package (Ritchie et al., 2015) was used to identify differentially expressed genes (DEGs) between the pediatric influenza group and the control group as disease targets in network pharmacology. A heatmap was generated based on these DEGs using R software’s pheatmap package.

### 2.3 Herb-component-target network construction and ImmuneGene analysis

To establish a scientifically sound relationship between compounds in herbs and their targets, we utilized Cytoscape 3.9.1 to construct an herb–component–target network that visually represented this relationship. We conducted a topological analysis to identify key components within this network. Immune related genes were collected through the ImmPort database (https://www.immport.org). The overlapping genes between immune related genes and BKN-DEGs are defined as BKN-ImmuneDEGs. Selection and visualization of BKN-ImmuneDEGs by using R packages “ggplot2” and “pheatmap”. Analyzing the gene network of BKN-ImmuneDEGs through the GeneMANIA database (https://genemania.org/).

### 2.4 PPI network analysis construction

To explore the core targets of BKN’s anti influenza effect, a protein-protein interaction (PPI) network was built using the STRING (https://cn.string-db.org/; Version 12.0) (Feng et al., 2019) database to examine the functional interaction between proteins, with setting the species to *H. sapiens* and network confidence score ≥0.4. Furthermore, the Cytohubba plug-ins were utilized to identify the core targets of BKN for combating pediatric influenza.

### 2.5 GO and KEGG pathway enrichment analysis

For the Kyoto Encyclopedia of Genes and Genomes (KEGG) and Gene Ontology (GO) enrichment analysis, we imported the gene symbols into the Metascape platform (http://metascape. org/), the organism used was *H. sapiens* (Zhou et al., 2019). The GO and KEGG pathway enrichment analysis findings were saved, and bioinformatics software (http://www.bioinformatics.com.cn/) and R version 4.3.1 finished the visualization.

### 2.6 Screening for BKN-related differentially expressed genes

Using R packages (such as “limma,” “pheatmap,” and “ggpubr”), we retrieved the expression levels of BKN-related genes from the influenza group and the normal group and performed differential expression analysis. Box plots and a heat map were utilized to present the results. BKN-related core genes (BKN-RCG) were classified as genes with a p-value less than 0.05. The BKN core genes were found on the chromosomes using perl code, and we then utilized the R package “Rcircos” to visualize them as circle plots. Additionally, using the “cor” command, correlation coefficients for each BKN-RCG were computed and displayed.

### 2.7 Unsupervised clustering and PCA of influenza-related BKN-RCG

We used the R package “ConsensusClusterPlus” to cluster influenza samples according to the expression of BKN-RCG with a k-means clustering method, Euclidean distance type, and maximum of nine clusters (Wilkerson and Hayes, 2010). Heat maps were used to compare the expression levels of the generated clusters. Lastly, PCA was used to illustrate the differences between the subtypes.

### 2.8 Weighted gene co-expression network (WGCNA)

To investigate the expression of gene sets, we utilized the weighted gene co-expression network analysis (WGCNA) approach. The R package “WGCNA” was employed to construct a co-expression network for DEGs. We selected the top 25% of genes with the highest variation to ensure biologically relevant gene modules and determined the soft-thresholding power based on the standard scale-free topology criterion (scale-free *R* ^2^ ≥ 0.9), which is a widely accepted approach (Langfelder and Horvath, 2008). By determining the optimal soft power, we generated a weighted adjacency matrix and transformed it into a topological overlap matrix (TOM). TOM is a matrix used to describe the degree of topological overlap between nodes in complex networks, which is widely used to analyze the characteristics such as the stability, robustness and community structure of the network. Larger TOM values indicate stronger stability and robustness of the network, while smaller TOM values indicate weaker stability and robustness of the network (Shuai et al., 2021). Using a hierarchical clustering tree technique with a minimum module size set at 100, we identified modules based on TOM difference measure (1-TOM). From these co-expression modules, we could extract key modules that exhibited high correlation with specific phenotypes or diseases. Hub genes were then identified based on internal connectivity within the key module and their correlation with feature vectors of the key module (Langfelder and Horvath, 2008).

### 2.9 Screening and validation of hub genes in pediatric influenza

We applied the “VennDiagram” program to identify intersected genes between DEGs, diseases, and clusters. Subsequently, we used three machine-learning algorithms to screen important biomarkers for pediatric influenza: RF (Ishwaran and Kogalur, 2010; Wang H. et al., 2016; Perera et al., 2020), LASSO logistic regression (Cheung-Lee and Link, 2019a; Fernández-Delgado et al., 2019), and SVM-RF (Huang et al., 2018a). The SVM algorithm identifies the optimal variables by deleting SVM-generated eigenvectors (Liu et al., 2023). LASSO utilizes regularization to reduce prediction errors (Yang et al., 2018). RF can handle high-dimensional data, establish predictive models, and predict the importance of each variable (Blanchet et al., 2020). Finally, the genes obtained by all three algorithms were ultimately crossed to screen for the characteristic genes. To validate the usefulness of these hub genes comprehensively, the dataset from GSE42026 served as our validation set. The predictive capability of these algorithms was evaluated using ROC curves, and area under curve (AUC)was calculated accordingly.

### 2.10 Molecular docking

We obtained the 3D structures of BKN-core components from Pubchem. And the 3D structure of feature genes was obtained through the AlphaFold Protein Structure Database (https://alphafold.com/). Initial conformations for each component were established using energy minimization techniques in Chem3D 14.0 software. AutoDockTools was utilized for molecular docking, with the best four combinations of docking selected and visualized using Pymol and Discovery Studio 2019.

### 2.11 Molecular dynamics (MD) simulation

MD simulation was performed on the receptor-ligand with the lowest binding energy from molecular docking results. GROMACS 2023.3 software was used to carry out MD simulations, utilizing amber14sb force field and general Amber force field (GAFF) to generate parameter and topology data for proteins and ligands respectively. The simulation box size was optimized based on protein atom distance greater than 1 nm, followed by water molecule filling at a density of one to achieve electrical neutrality through Cl^−^ and Na^+^ ion replacement methods. Using the steepest descent approach, 5.0 × 10^4^ steps of energy optimization were carried out to minimize the overall system’s energy consumption and, finally to lower the overall system’s unreasonable contact or atom overlap. Then, the system’s temperature was stabilized through first-phase equilibration using the NVT ensemble at 300 K for 100 ps. The NPT ensemble was used to mimic second-phase equilibration at 100 ps and 1 bar. To thoroughly pre-equilibrate the simulation system, the main goal of the simulation is to optimize the interaction between the target protein and the solvent and ions. MD simulation ran for 50 ns at a temperature of 310.15 K and 1 atm of pressure in an isothermal and isostatic ensemble. The V-Rescale and C-Rescale techniques were used to regulate the temperature and pressure, respectively. The temperature and pressure coupling constants were 0.1 ps. The Van der Waals force was computed using the Leonard-Jones function, and the nonbond truncation distance was established at 1.4 nm. The LINCS algorithm limited the bond length of every atom. Using a 0.16 nm Fourier spacing and the Particle Mesh-Ewald method, the long-range electrostatic interaction was computed. Finally, we used gmx_mmpbsa to calculate the binding free energy of the compound (http://jerkwin.github.io/gmxtool). Notably, to further demonstrate the stability of the binding between the small molecule and the protein, all MD simulations were performed twice. In the data presentation, the RMSD, RMSF, Rg, H-bond, and SASA metrics represent the mean values from the two simulations, while the MMPBSA analysis was conducted on the segments of the trajectories that exhibited greater stability.

## 3 Result

### 3.1 Collection of active ingredients and targets of BKN

The BKN active components were screened using the TCMSP database. Twenty prospective BKN compounds, eight from Pingbeimu, eight from Qingdai, and four from Baiguoren, were ultimately gathered (Supplementary Table S2). PharmMapper and UniProt databases were used to predict potential targets. Finally, 200 targets in Pingbeimu, 250 targets in Qingdai, and 229 targets in Baiguoren were collected.

### 3.2 Identification of DEGs in pediatric influenza from GEO

**FIGURE 2:**
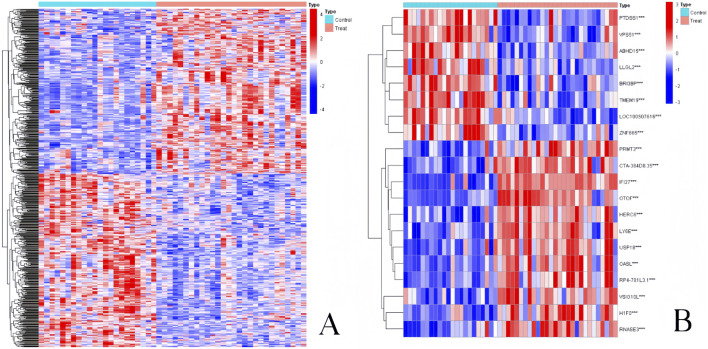
Heatmap of DEGs in pediatric influenza. **(A)** Heatmap of the entire DEGs. **(B)** Heatmap of the top 10 DEGs.

By computing the difference in gene expression between 22 normal samples and 28 influenza infection samples, we were able to select DEGs of pediatric influenza. Ultimately, the limma package in R software was used to identify 3,819 DEGs from the GSE34205 dataset. Figure 2A shows that 1747 upregulated and 2072 downregulated genes were found. Figure 2B displays the top 10 genes with the greatest up- and downregulation.

### 3.3 Herb-component-target network construction and ImmuneGene analysis

**FIGURE 3:**
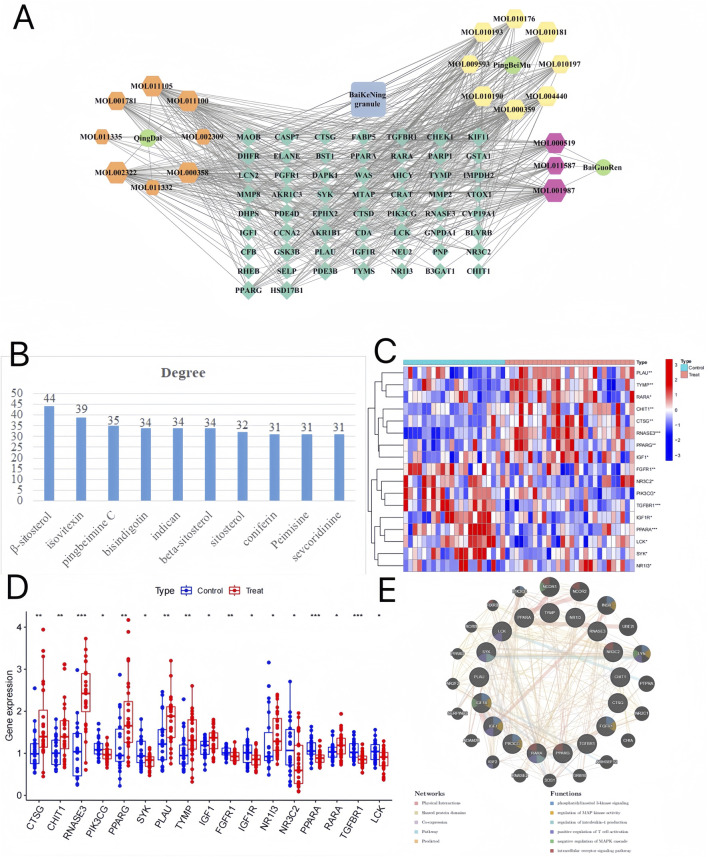
BKN-Herb-component-target network. **(A)** There were 3 kinds of herbs (Ping-Bei-Mu, Qing-Dai, Bai-Guo-Ren), 19 compounds, and 42 related targets on the network. The blue square represents BKN. Hexagons of different colors represent the active ingredients of different medicinal herbs. The dark green diamond represents the intersection of drug and disease targets. Green represents medicinal herbs. **(B)** According to the BKN-Herb-component-target network, the degree values of different compounds rank in the top 10. **(C, D)** The box plot and heatmap showed expression patterns of BKN-ImmuneDEGs in pediatric influenza. **(E)** Gene network and functional analysis of ImmuneCDEGs generated using GeneMANIA. The inner circle contains ImmuneCDEGs, while the outer circle contains reciprocal genes. *p < 0.05, **p < 0.01, ***p < 00.001.

58 potential targets of BKN in combating pediatric influenza were shown in Supplementary Table S3. We utilized Cytoscape and its plugins to construct “herb-component-target” network, with 79 nodes and 497 edges (Figure 3A). β-sitosterol was the most prolific, with 42 potential targets, secondly isovitexin (38), and pingbeimine C (34), bisindigotin, indican, beta-sitosterol, sitosterol, coniferin, Peimisine, and sevcoridinine as illustrated in Figure 3B. The course of influenza disease is closely related to immune activity. Therefore, we further investigated the correlation between immune related genes and selected genes that overlap with BKN related DEGs as BKN-ImmuneDEGs. A total of 17 BKN ImmuneDEGs were obtained, including nine upregulated genes (CTSG, CHIT1, RNASE3, PPARG, PLAU, TYMP, IGF1, NR1I3, and RARA) and eight downregulated genes (PIK3CG, SYK, FGFR1, IGF1R, NR3C2, PPARA, TGFBR1, and LCK), as illustrated in Figures 3C,D. Further import these BKN-ImmuneDEGs into GeneMANIA to construct a gene network (Figure 3E). The gene mainly involved in response to phosphatidylinositol 3-kinase signaling, regulation of MAP kinase activity, regulation of interleukin-4 production, positive regulation of T cell activation, negative regulation of MAPK cascade, and intracellular receptor signaling pathway.

### 3.4 Analysis of protein-protein interaction networks

**FIGURE 4:**
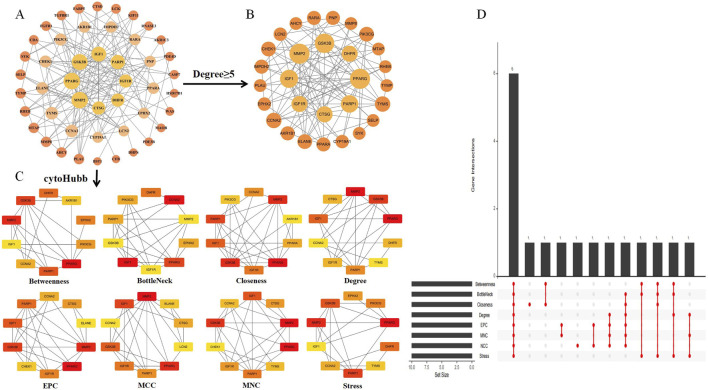
Intersection target PPI network and network analysis. **(A)** Intersection target PPI network. The orange circle represents the intersection target, with a larger degree value indicating a larger shape. **(B)** Intersection targets greater than or equal to the standard degree value. **(C)** The CytoHubba plugin was used to identify the core targets from the PPI network. The node’s color ranged from pale yellow to red, with a matching increase in degree. **(D)** The Upset graph shows the results of eight algorithms represented as core targets.

Using the intersection targets from Cytoscape and STRING, we built a PPI network with 144 edges and 47 nodes, with an average node degree of 4.97 (Figure 4A). A p-value of less than 1.0e-16 indicated significant clustering in the PPI network. Twenty-nine key genes were initially filtered out of the data based on degree values larger than or equal to the standard degree value (Figure 4B). Then, we used eight random algorithms from the CytoHubba plugin to further determine the top 10 central genes, and identified the core targets through intersection, as illustrated in Figure 4C. We use R to draw an Upset graph to display the results of each algorithm. The core targets include PPARG, MMP2, GSK3B, PARP1, CCNA2, and IGF1, as illustrated in Figure 4D.

### 3.5 Functional enrichment analysis of target

**FIGURE 5:**
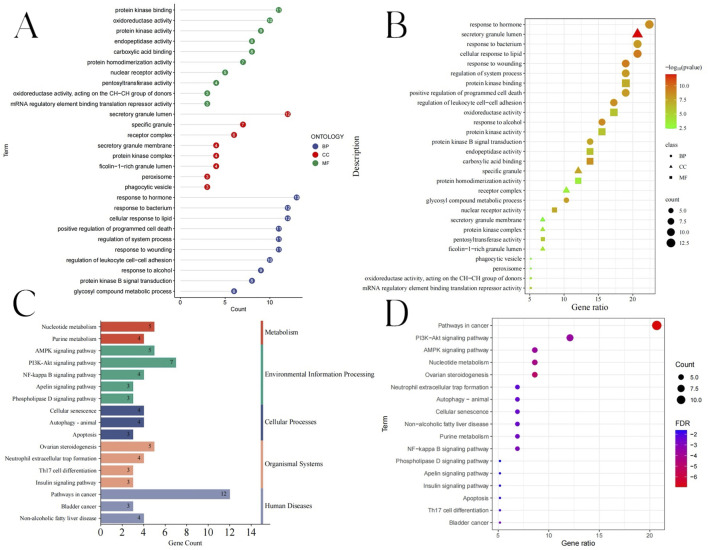
GO and KEGG enrichment analysis results. **(A)** Lollipop diagram showing the BP, CC, and MF. **(B)** Bubble chart showing the BP, CC, and MF. **(C)** The KEGG enrichment analysis bubble chart displaying the top 17 pathways. **(D)** The top 17 pathways’ KEGG types, as determined by KEGG enrichment analysis.

Based on biological process (BP), cellular component (CC), and molecular function (MF), GO enrichment analysis was examined. To further investigate the various mechanisms by which BKN contributes to pediatric influenza treatment, we ran a GO enrichment analysis of the 58 common targets. Three groups containing a total of 521 items were found: 445 BP, 48 MF, and 28 CC (Supplementary Table S4). As bubble charts and lollipop charts, the top 10 enriched BP terms, MF terms, and CC terms are displayed (Figures 5A,B). Result showed that the BP terms were mainly involved the cellular response to lipid, response to wounding, response to alcohol, response to hormone, and regulation of leukocyte cell-cell adhesion. Highly enriched MF terms were carboxylic acid binding, nuclear receptor activity, protein kinase binding, endopeptidase activity, and oxidoreductase activity. In addition, the CC terms included secretory granule lumen, specific granule, ficolin-1-rich granule lumen, and protein kinase complex. We evaluated KEGG pathway enrichment analysis of 58 common targets (with P < 0.05 as the significance level). 52 pathways had targets that were substantially enriched (Supplementary Table S5). Gene counts were used to filter the top 17 significantly enriched pathways (Figures 5C,D). KEGG enrichment results showed that nucleotide metabolism, AMPK signaling pathway, PI3K-Akt signaling pathway, ovarian steroidogenesis, and pathways in cancer.

### 3.6 BKN-related core genes expression difference, chromosome position, and expression correlation analysis

**FIGURE 6:**
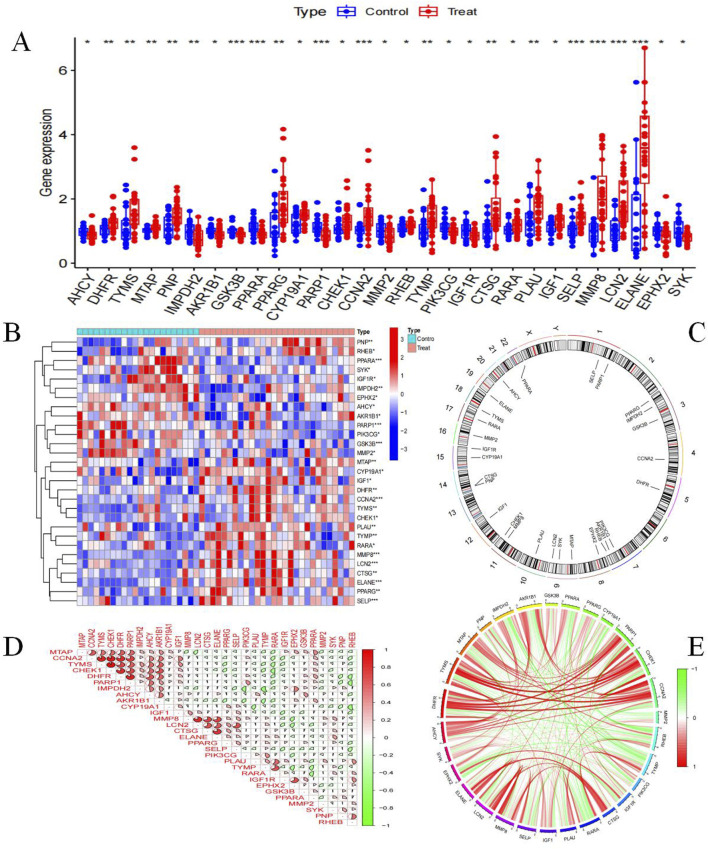
Analysis of BKN-RCG. **(A)** Box plot of core gene expression differential analysis between normal samples and pediatric influenza samples, (*p < 0.05, **p < 0.01, ***p < 0.001). **(B)** Expression of BKN-RCG in normal and pediatric influenza. **(C)** Chromosome location of BKN-RCG. **(D)** Correlation analysis between the two BKN-RCG. **(E)** BKN-RCG correlation network.

We obtained 29 BKN core genes through network pharmacology analysis (Supplementary Table S6). To further analyze the network pharmacology results, we obtained 29 BKN-RCG by intersecting the BKN target with DEGs. Among these genes, a total of 18 genes are highly expressed in pediatric influenza, and a total of 11 genes are highly expressed in the normal group (Figures 6A,B). Figure 6C shows the specific chromosomal locations of the BKN-RCG. As shown in Figures 6D,E, correlation analysis between every two BKN-RCG in pediatric influenza samples revealed a substantial correlation between the BKN-RCG, with the correlation being predominantly positive.

### 3.7 Identification of BKN-related molecular clusters in influenza

**FIGURE 7:**
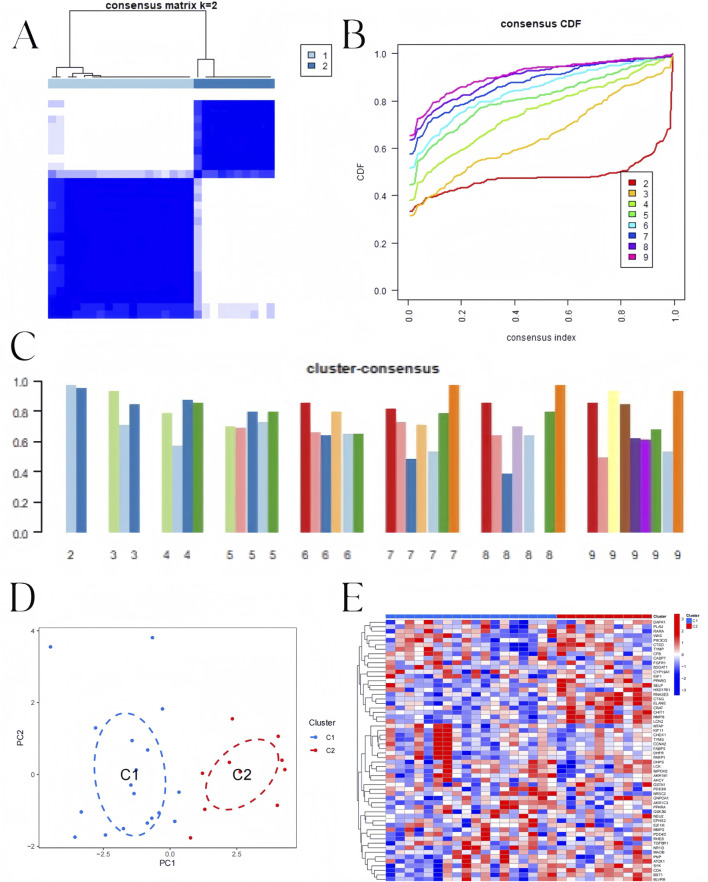
BKN-related clusters in pediatric influenza. **(A)** Matrix of consensus at k = 2. **(B)** Cumulative distribution function (CDF). **(C)** The consensus clustering score. **(D)** The two subclusters’ distribution in PCA. **(E)** Heatmap showing the 58 BKN-dysregulated between the two clusters.

To identify BKN-related molecular clusters in influenza. We used all 58 intersection genes related to influenza in BKN. Studies have indicated that the most stable and credible clustering findings (Figure 7A) and cumulative distribution function (CDF) curves fluctuating within a minimum range (Figure 7B) were obtained when k = 2, and the consistency score was greater than 0.9 (Figure 7C). A significant difference between the two clusters was found using principal component analysis (PCA) (Figure 7D). The expression differences of BKN-related intersection genes between Cluster 1, Cluster 2 as illustrated in Figure 7E.

### 3.8 Identification of significant module genes in pediatric influenza via WGCNA and cluster WGCNA

**FIGURE 8:**
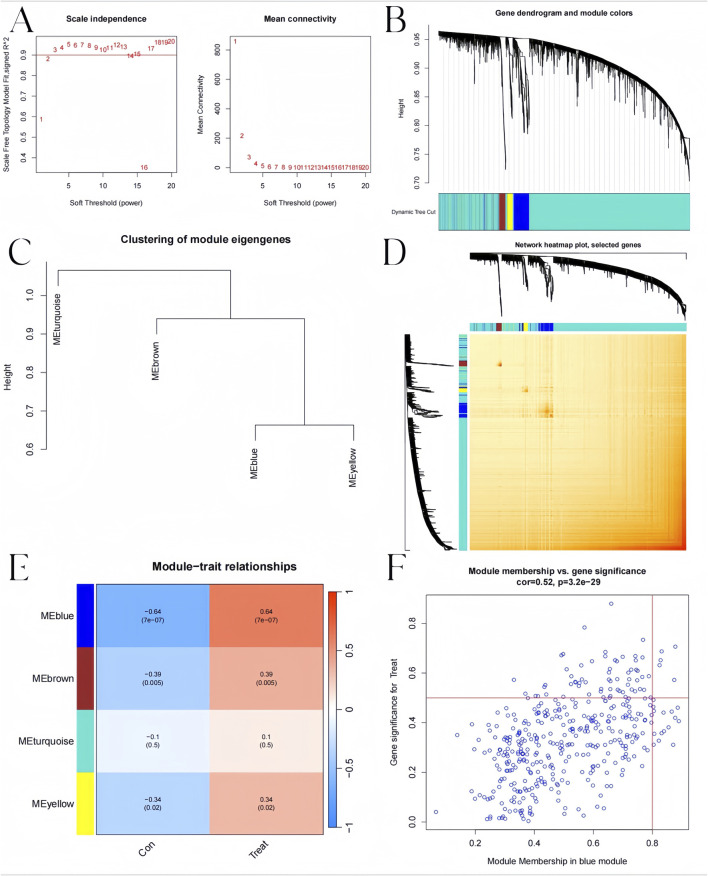
Co-expression network in pediatric influenza. **(A)** Soft threshold selection. **(B)** Dendrogram of genes within the co-expression module. Various modules are displayed using various colors. **(C)** A map of feature genes’ clustering within modules. **(D)** Correlation heatmap between modules. **(E)** Module signature gene correlation analysis with clinical traits. **(F)** Gene significance for pediatric influenza and blue module genes is plotted together in scatter plots.

We constructed the co-expression network through WGCNA package for identifying significant module genes of pediatric influenza. Grouping genes with comparable expression patterns into the same gene module through average linkage clustering. We screened the top 25% of genes with the highest fluctuations for WGCNA analysis. The results showed that when the soft threshold was 3 and the scale-free R2 was 0.9, we identified co-expressed gene modules (Figure 8A). A total of four distinct gene co-expression modules were generated via WGCNA analysis while showing the heatmap of the TOM (Figures 8B–D). The module with the smallest p-value is the one most related to the disease. The WGCNA result shows that the blue module was highly related to pediatric influenza (Figure 8E). Meanwhile, the blue module was positively correlated with module-associated genes (Figure 8F).

**FIGURE 9:**
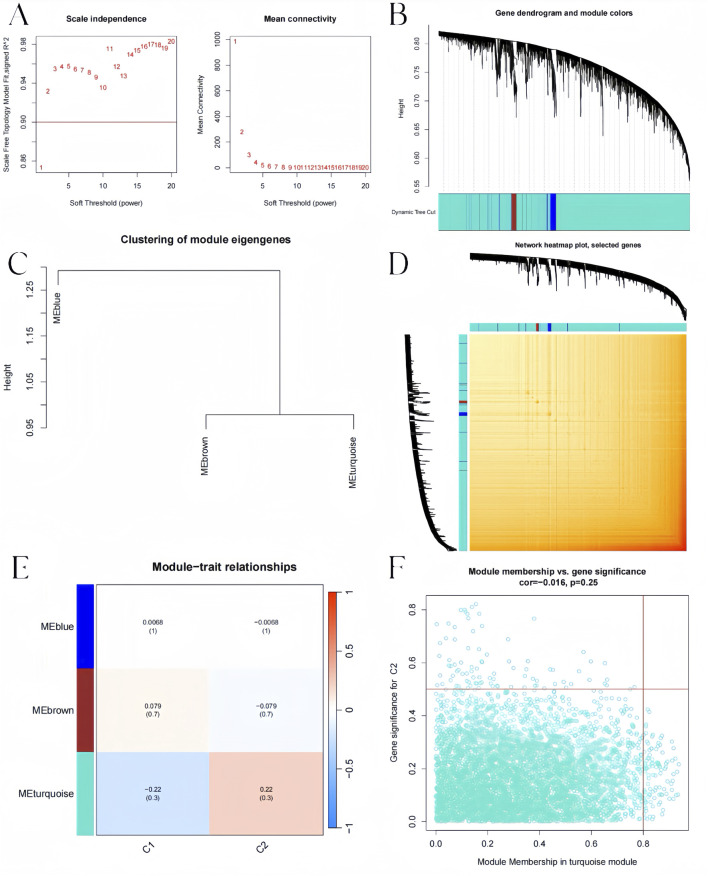
BKN-related molecular clusters’ co-expression network in pediatric influenza. **(A)** Soft threshold selection. **(B)** Dendrogram of genes within the co-expression module. Various modules are displayed using various colors. **(C)** A map of feature genes’ clustering within modules. **(D)** Correlation heatmap between modules. **(E)** Module signature gene correlation analysis with clinical traits. **(F)** Gene significance for pediatric influenza and the turquoise module genes is plotted together in scatter plots.

Additionally, the WGCNA algorithm was used to generate gene modules and co-expression networks for three BKN-related molecular clusters to identify the critical gene modules linked to pediatric influenza. The results revealed the co-expression gene modules when the scale-free R2 was 0.9 and the soft threshold was 3 (Figure 9A). While displaying the TOM heatmap, the dynamic cutting algorithm produced three co-expression modules in various hues (Figures 9B–D). When the genes within the three modules were compared to ascertain the degree of gene co-expression similarity between influenza and controls, the 5119 genes in the turquoise module were most strongly linked to the BKN-related molecular cluster (Figure 9E). Moreover, the turquoise module showed a high correlation with genes related to modules (Figure 9F).

### 3.9 Candidate hub genes of pediatric influenza selection and validation

**FIGURE 10:**
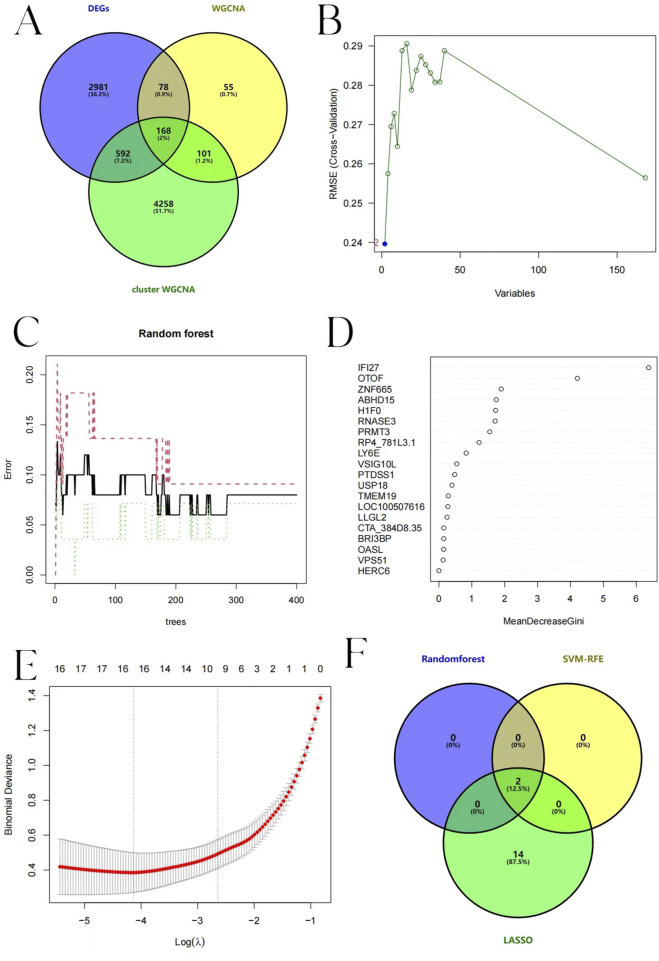
Candidate hub pediatric influenza genes selection. **(A)** Venn diagram showing the gene that connects the influenza module-related genes to the BKN-related molecular clusters module-related genes. **(B)** Biomarker screening based on SVM-RFE. The horizontal axis represents the number of retained variables, and the vertical axis represents RMSE. **(C)** Error variation trend of RF. The black solid line represents the overall error. The red dotted line and the green dotted line respectively represent the error rates of different classification results. **(D)** MeanDecreaseGini of each variable in RF algorithm. **(E)** LASSO logistic regression algorithm to screen biomarkers. The horizontal axis represents Log(λ), and the vertical axis represents Binomial Deviance. The red dotted line represents the λ value corresponding to the point where the deviation is the smallest, and the number above it is the number of variables corresponding to each λ value. **(F)** A Venn diagram displaying the points where the three algorithms’ diagnostic markers intersected.

Taking the intersection of DEGs, WGCNA, and BKN-related cluster WGCNA, we identified 168 key genes (Figure 10A) and performed further exploration of them. Lasso regression, RF, and SVM-RFE algorithms were adopted to screen candidate hub genes for ROC evaluation. According to the SVM-RFE algorithms display N = 2, Root Mean Squared Error (RMSE) (0.24) is the smallest (Figure 10B). This indicated that the model performance was optimal under this combination of features. Therefore, the two optimal candidate genes were screened out from SVM-RFE. The relationship between the error rate, the number of classification trees, and the two genes in descending order of relative relevance was found using RF in conjunction with feature selection (Figures 10C,D). Interferon alpha-inducible protein 27 (IFI27) and Otoferlin (OTOF) rank first and second respectively, suggesting their significance in classification tasks. The optimal λ value is selected by using 10-fold cross-validation to achieve feature selection. And 16 predicted genes were chosen using LASSO regression analysis from the set of statistically significant univariate variables. Figure 10E illustrates the Lasso regression results. The three algorithms identified IFI27 and OTOF as hub genes with overlap (Figure 10F).

**FIGURE 11:**
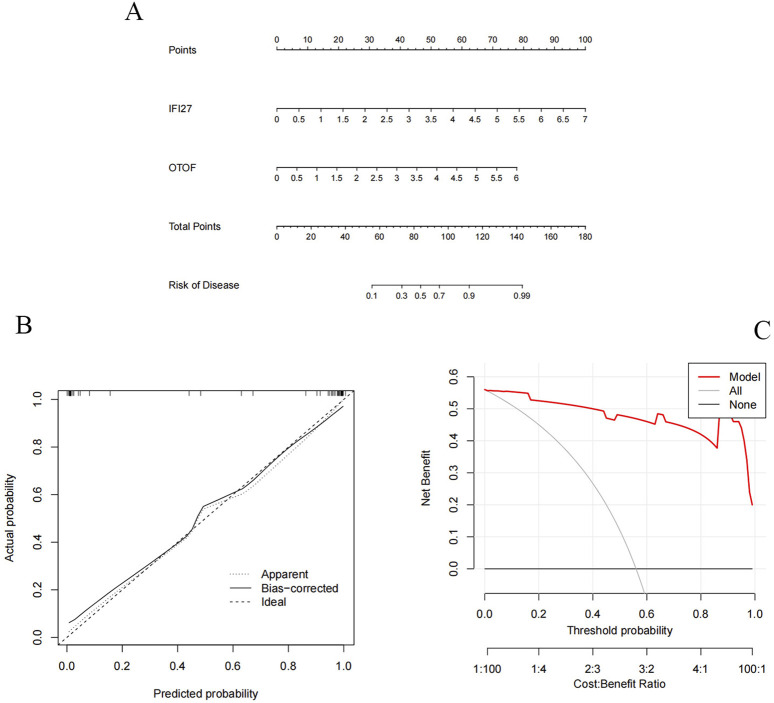
Candidate hub genes of pediatric influenza validate. **(A)** Nomogram shows the incidence rate of pediatric influenza. **(B)** Correction curve of characteristic gene column chart for influenza A. **(C)** Decision curve of feature genes nomogram of pediatric influenza.

**FIGURE 12:**
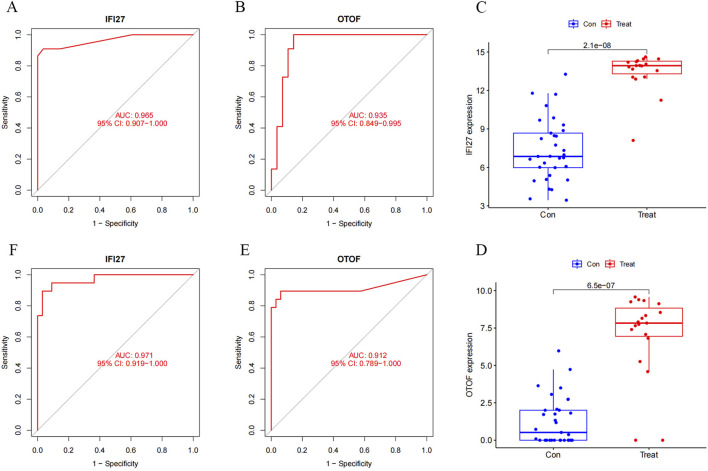
Candidate hub genes of pediatric influenza validate by ROC and expression. **(A)** ROC of the IFI27 in experimental set. **(B)** ROC of the OTOF in experimental set. **(C)** Boxplot of the IFI27. **(D)** Boxplot of the OTOF. **(E)** ROC of the IFI27 in validation set. **(F)** ROC of the OTOF in validation set.

We constructed a column chart using IFI27 and OTOF to obtain individual score tables (Figure 11A). The total of these distinctive genes’ expression scores can be used to calculate treatment sensitivity and forecast the risk rate of these genes in the development of pediatric influenza. Two indicators of the accuracy of this prediction are the proximity of the dashed and solid lines in the calibration curve (Figure 11B) and the distance between the red and gray lines in the decision curve (Figure 11C). The ROC curves for IFI27 and OTOF (Figures 12A,B) demonstrated their likelihood as important biomarkers with AUCs of 0.965 and 0.935, respectively. This suggests that the biological markers had a good predictive value accuracy. Within the GSE42026 validation set, there was a significant difference (P < 0.01) in the expression of IFI27 and OTOF between the pediatric sepsis and control groups (Figures 12C,D). The ROC curves for IFI27 and OTOF, with AUCs of 0.971 and 0.912, respectively, suggested that their likelihood as valuable biomarkers in the GSE42026 validation set (Figures 12E,F).

### 3.10 Molecular docking validation

**FIGURE 13:**
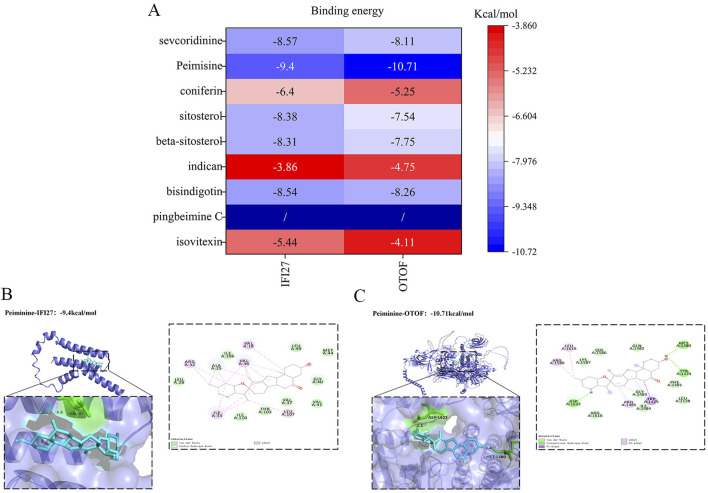
Molecular docking validation. **(A)** Heat map showing the results of the molecular docking between hub genes and active components. **(B)** Peimisine- IFI27 docking models. **(C)** Peimisine-OTOF docking models.

We conducted molecular docking to investigate whether the proteins encoded by the pediatric influenza hub genes we obtained can bind and function with the active components of BKN. The protein structures of IFI27 (AF-P40305-F1-v4) and OTOF (AF-Q9HC10-F1-v4) were downloaded from the AlphaFold protein structure database. The results indicate that the hub genes and active components could form stable structures, and the binding energy of most docking combinations is lower than −5.0 kcal/mol (Figure 13A). This implies that most of the hub genes and active components could form stable structures. The best docking combinations (Peimisine- IFI27 and Peimisine-OTOF) are shown in Figures 13B,C.

### 3.11 Molecular dynamics simulation

In view of the results of molecular docking, the following MD studies mainly focus on the relationship between Peimisine-IFI27 and Peimisine-OTOF, respectively. MD reveals the stability of protein-ligand complexes in physiological aqueous solutions at 37°C (310.15 K) and *in silico* under standard atmospheric pressure (a pressure of 1 atm). To assess their performance, we used Peimisine-OTOF and Peimisine-IFI27 for 50 ns dynamics simulations. We ran the molecular dynamics simulation twice to ensure reproducibility. And the standard deviation was used to represent the accuracy of repetitive measurements. The results showed that the value of the standard deviation can prove that the results in this study had excellent reproducibility. The detailed results of the two simulations can be found in Supplementary Tables S7 and S8. The initial and final structures of every trajectory in PDB format can be found in Supplementary Material.

**FIGURE 14:**
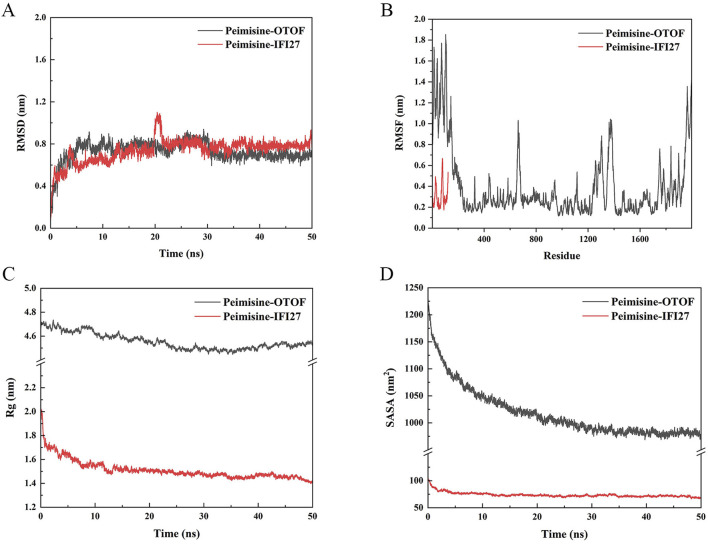
Molecular docking validation. **(A)** The RMSD of Peimisine- IFI27 and Peimisine-OTOF. **(B)** The RMSF of Peimisine- IFI27 and Peimisine-OTOF. **(C)** The Rog of Peimisine- IFI27 and Peimisine-OTOF. **(D)** The fluctuation plot of the complexes SASA.

It can be observed that all two complexes showed good stability, the average RMSD values for Peimisine- IFI27 and Peimisine-OTOF are as follows: 0.737 nm and 0.729 nm. After 25 ns of MD simulation, the RMSD-time curves became steady (Figure 14A), and the RMSD value of Peimisine-IFI27 was stable at 0.8 nm, while the Peimisine-OTOF was stable at 0.7 nm. In general, the system is said to be stable once the RMSD value stabilizes, and the fluctuation range is smaller than 0.2 nm (Saranya et al., 2023). The majority of the atomic RMSF values varied between 0.2 and 1.2 nm during the MD simulation, the average RMSF values for Peimisine-IFI27 and Peimisine-OTOF are as follows: 0.304 nm and 0.386 nm. Additionally, a nearly horizontal line was created by the Rg values, indicating a relatively stable complex system, the average Rg values for Peimisine-IFI27 and Peimisine-OTOF are as follows: 1.512 nm and 4.555 nm. (Figures 14B,C). Moreover, the Rg values of Peimisine- IFI27 were smaller than Peimisine-OTOF. In conclusion, these findings demonstrate the superior conformational stability of those two complexes and provide compelling evidence for the validity of docking. In addition, to further evaluate the binding interactions in protein-ligand complexes, we quantified the solvent-accessible surface area (SASA) to indicate changes in molecular conformation or exposure during the simulation process. The average SASA values for complexes Peimisine- IFI27 and Peimisine-OTOF during MD simulation are as follows: 73.792 nm^2^ and 1018.103 nm^2^. The SASA values exhibited minor fluctuations throughout the course of the simulation, demonstrating their stable interaction (Figure 14D).

To determine the strength and stability of protein-ligand interactions, we computed the difference between the free energies of bound and unbound states utilizing Molecular Mechanics–Poisson Boltzmann Surface Area (MMPBSA). We selected 10 ns trajectories to choose segments with steady RMSD values for MMPBSA energy analysis. The sum of the entropy (–TΔS) and enthalpy (ΔH) changes was the total binding energy (Wilson Alphonse and Kannan, 2023). We utilized the gmx_mmpbsa.bsh script to evaluate the entropy change in each complex at intervals of 1000 ps. The total binding energy was dissected into four distinct components as shown in Table 1. Table 1 presents the outcomes of protein-ligand binding energies. In the system comprising protein-ligand complexes, Peimisine-OTOF and Peimisine-IFI27 proteins exhibited negative binding free energies with small molecules (−126.691 kJ/mol and −125.930 kJ/mol respectively), indicating a more stable association between Peimisine and IFI27. Notably, Van der Waals interaction played a significant role in this main energy interaction.

**TABLE 1:** MMPBSA analysis of proteins and small molecules in complex simulation processes.

Energy	Peimisine-OTOF	Peimisine-IFI27
Van der Waals Energy (KJ/mol)	−212.534	−145.503
Electrostatic energy (kJ/mol)	−0.306	−11.432
Polar solvation energy (KJ/mol)	97.126	39.728
Nonpolar solvation Energy (KJ/mol)	−29.374	−21.871
Total Binding Energy (KJ/mol)	−145.089	−139.079
-T∆S(KJ/mol)	18.397	13.149
Total Binding Free Energy (KJ/mol)	−126.691	−125.930

**FIGURE 15:**
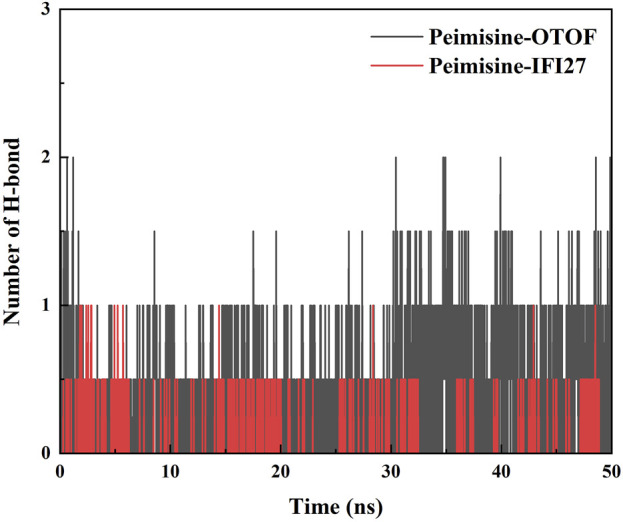
The hydrogen bonds of Peimisine- IFI27 and Peimisine-OTOF.

Finally, we determined the change in the alteration in hydrogen bond count within the two complexes throughout the 50 ns simulation process. Hydrogen bonds served as indicators of the affinity between the ligand and protein. The docked complex exhibited a stable pattern of hydrogen bonds (Figure 15) (Cong et al., 2023). Our findings indicated that there is fluctuation in the number of hydrogen bonds within both complexes, ranging from 0 to 2. The average count of hydrogen bonds observed during MD simulation for Peimisine-IFI27 and Peimisine-OTOF are as follows: 0.041 and 0.346 respectively.

**FIGURE 16:**
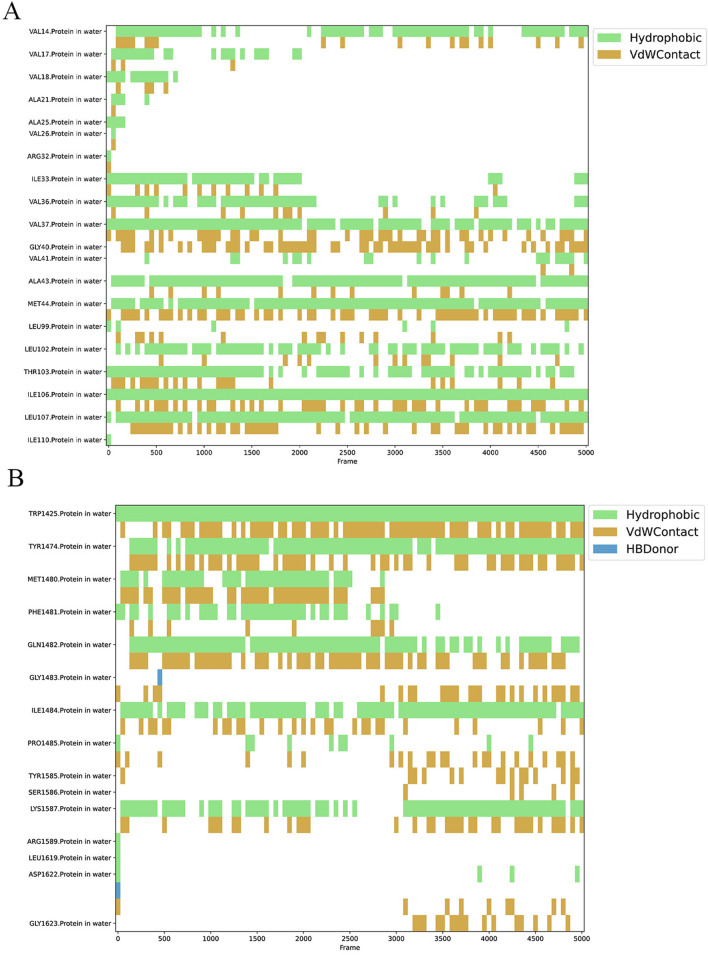
Key contacts and their residues analyzed in Peimisine-IFI27 complex **(A)** and Peimisine-OTOF complex **(B)**.

To demonstrate the stability of the binding between the small molecule Peimisine and two proteins during the MD process, the python package Prolif and MDAnalysis was employed to assess the bonding interactions between the small molecules and the proteins throughout the MD simulations (Figures 16A,B). The results indicated that in the IFI27 protein, the residues Val14, Val37, Ala43, Met44, Leu102, Thr103, Ile106, and Ile110 maintained hydrophobic and van der Waals interactions with the small molecule for most of the simulation time. In contrast, in the OTOF protein, the residues Trp1325, Tyr1474, Gln1482, Ile1484, and Lys1587 exhibited similar stable hydrophobic and van der Waals interactions with the small molecule throughout most of the MD simulation process. The stable interactions of these residues further corroborate that Peimisine maintains a stable binding with either the IFI27 or OTOF protein throughout the MD process.

## 4 Discussion

Children around the world are greatly affected by influenza, resulting in high hospitalization rates and significant morbidity and mortality (Nayak et al., 2021). The high risk of viral variability, drug resistance, and drug development leads to a scarcity of drugs for treating viral diseases (Xiong et al., 2020). TCM has a successful history of treating infectious diseases, including influenza which is classified as “YI Disease” according to TCM theory. This classification was first reported in Huang Di Nei Jing (Li et al., 2021). In modern times, TCM has been successfully used to manage various pandemics, including SARS-CoV in 2003, MERS-CoV in 2012, seasonal epidemics caused by influenza viruses and dengue viruses, and SARS-CoV-2 (Huang et al., 2021). The marketed TCM BKN is mainly used for lung heat, phlegm heat, asthma syndrome, and *pertussis* in children, etc. It has the effects of clearing the liver, purging fire, promoting lung function, relieving asthma, and relieving cough and phlegm. Due to the different reactions of children to drug metabolism compared to adults, expanding the therapeutic applications of marketed TCM for children may be an effective way to explore new drugs. Given the therapeutic effect of BKN, we attempt to explore whether BKN can be used to treat pediatric influenza.

BKN is composed of three kinds of TCMs: Ping-Bei-Mu, Qing-Dai, and Bai-Guo-Ren. Studies have found that Qing-Dai can alleviate lung inflammation, downregulate pro-inflammatory cytokines, and inhibit the JAK2/STAT3 signaling pathway (Qi et al., 2024). Ping-Bei-Mu can inhibit the recruitment of inflammatory cells and the production of cytokines, and treat respiratory inflammation (Wang D. et al., 2016). The extract of Bai-Guo-Ren has been proven to inhibit pulmonary fibrosis (Daba et al., 2002). The possible mechanism of BKN in pediatric influenza was systematically in this study using network pharmacology techniques. By means of the “drug-component-target” network analysis, the primary material basis for BKN’s therapeutic actions on pediatric influenza had been determined. We found that β-sitosterol was the compound with the highest pharmacological activity in BKN treatment of pediatric influenza. It had been demonstrated that β-sitosterol, a common phytosterol found in Chinese medicinal plants, had anti-inflammatory and antioxidant properties. Research has found that β-sitosterol had promising antiviral activity against H1N1 virus, as it can reduce virus titers in a concentration dependent manner and exert its anti-influenza effect (Shokry et al., 2023). β-sitosterol can block retinoic acid-inducible gene I (RIG-I) signaling, and immune responses mediated by harmful interferons (IFNs) production, providing potential benefits for the treatment of influenza (Zhou et al., 2020). Isovitexin is the main flavonoid compound of Vigna radiata extract (VRE). VRE has been found to inhibit the entry of the influenza virus by directly blocking the HA protein of the influenza virus (Lo et al., 2020). Isovitexin has been proven to interact with the human angiotensin-converting enzyme 2 (hACE2) receptor and possesses hACE2 receptor blocking properties (Ferdausi et al., 2022). Another study found through virtual screening that bisindigotin may interrupt the interaction between the hACE2 receptor and the SARS-Cov-2 viral spike protein (Wei et al., 2020). Peimisine is one of the main alkaloids of Ping-Bei-Mu. Studies have found that Peimisine can inhibit oxidative stress, DNA damage, apoptosis and autophagy dysregulation through the NRF2/KEAP1 and JNK/MAPK-dependent pathways, thereby slowing down the pathological progression of lung diseases (Liu et al., 2024). Peimisine can also alleviate the destruction of alveolar structure and reduce the aggregation of inflammatory cells, thereby preventing pulmonary interstitial fibrosis (Jiao et al., 2024). In addition, Peimisine can increase the content of IκB protein, reduce the content of p65 protein in lung tissue, and exert anti-lung injury by inhibiting the activity of the NF-κB signaling pathway (Jin et al., 2022).

We analyzed six hub genes, including PPARG, MMP2, GSK3B, PARP1, CCNA2, and IGF1, using the PPI network combined with CytoHubba plugin. Moreover, GO and KEGG analysis of 58 targets showed that BKN may prevent pediatric influenza through nucleotide metabolism, AMPK signaling pathway, PI3K/Akt signaling pathway, and cellular response to lipids. Research had found that the AMPK signaling pathway was associated with influenza A virus (IAV) replication and IAV pneumonia. Activating the PPARG/AMPK pathway could significantly protect mice from IAV infection (Bei et al., 2021). The activation of the PI3K/Akt signaling pathway occurred through various mechanisms in a biphasic manner and played multiple roles during IAV infection. Phosphatidylinositol-3-kinase (PI3K) could regulate early step during viral entry, resulting in suppression of premature apoptosis at later stages of infection, and regulating the polarization of alveolar macrophages towards M1/M2b (Ehrhardt and Ludwig, 2009; Zhao et al., 2014). These pathways are associated with immune responses. Therefore, we further collected immune genes to study the immune related genes and pathways of BKN treatment for childhood influenza. A total of 17 BKN ImmuneDEGs were identified, mainly involving response to photosensitivity 3-kinase signaling, regulation of MAP kinase activity, regulation of interleukin-4 production, positive regulation of T cell activation, negative regulation of MAPK cascade, and intracellular receptor signaling pathway.

Secondly, a series of bioinformatics analyses were conducted based on gene expression profiles obtained from the GSE34205 dataset to identify 3,819 DEGs between pediatric influenza and normal samples. Based on these DEGs, we further combined WGCNA, BKN related cluster WGCNA, and three machine learning algorithms (LASSO, SVM RFE, and RF) to screen and identify IFI27 and OTOF as pediatric influenza hub genes associated with BKN. The RF model serves as an illustration of a supervised non-parametric technique employed for achieving classification (Zhang et al., 2023). LASSO identifies variables by seeking values that minimize the likelihood of classification errors (Cheung-Lee and Link, 2019b). SVM-RFE can sort features and select the most important features for classification (Huang et al., 2018b). The results of in-depth verification indicated that the hub genes IFI27 and OTOF found in children’s influenza were accurate. IFI27 belongs to the interferon-induced protein 12 family and is a nuclear encoded mitochondrial protein rich in brown fat tissue. Multiple clinical studies had found the value of IFI27 as a biomarker in various respiratory virus infections, including severe acute respiratory syndrome coronavirus type 2 (SARS-CoV-2) and respiratory syncytial virus (Tang et al., 2017; Gao et al., 2021; Shojaei et al., 2023). Villamayor et al. found through cellular and animal models that IFI27 protein levels were significantly upregulated in three unrelated viral infections caused by IAV, SARS-CoV-2, and Sendai virus (SeV). Using IFI27 knockout cells, it was found that IFI27 expression is positively correlated with virus production, possibly because it can counteract host induced antiviral responses, including *in vivo* antiviral responses (Villamayor et al., 2023). Another study found revealed the significant role played by IFI27 in cristae morphogenesis, ensuring the preservation of succinate dehydrogenase function and active fatty acid oxidation to sustain thermogenesis in brown adipocytes (Cui et al., 2023). OTOF had been found to be associated with neuronal transmission function, and in a mouse model with complete knockout of the OTOF gene, it was found that the auditory phenotype exhibited extremely severe deafness (Roux et al., 2006). Further research had found that downregulation of OTOF protein levels can lead to hearing loss (Strenzke et al., 2016). Meanwhile, Short et al. found that infection with influenza virus could lead to middle ear inflammation and hearing loss (Short et al., 2011). Our study found that IFI27 and OTOF protein levels were significantly reduced in children with influenza. Finally, we used molecular docking to validate β-sitosterol, bisindigotin, beta sitosterol, sitosterol, coniferin, Peimisine, and sevcodinine could bind to IFI27 and OTOF. Molecular dynamics further confirmed the stable binding of Peimisine- IFI27 and Peimisine-OTOF. This suggests that this may be the biological implications of BKN’s antiviral, mitochondrial metabolism, and thermogenic effects in treating pediatric influenza and its associated hearing loss.

In this investigation, we attempted to identify hub genes of pediatric influenza associated with BKN and further explore the role of BKN in pediatric influenza. Based on various data of “drugs”, “diseases”, and “children”, this study extensively examined the mechanism of BKN treatment for pediatric influenza from various perspectives such as genes, proteins, pathways, etc. utilizing network topology, WGCNA, machine learning, molecular docking, and MD methods. Previous studies on network pharmacology of TCM or herbal medicine for pediatric influenza treatment did not conduct thorough analysis of human samples. Instead, they relied on animal samples or simply extracted disease targets from datasets (Lai et al., 2020; Tang et al., 2022). Previous machine learning research had mainly been used to predict influenza virus genotypes to phenotypes (Borkenhagen et al., 2021), screen and identify genetic biomarkers (Chen et al., 2024), and research on childhood influenza was limited. This investigation took the target as the link and explored the multifaceted mechanisms of BKN treatment for pediatric influenza from the perspective of drug intervention. Therefore, compared with other studies, our research is more comprehensive and provides greater guidance in the exploration of novel drugs. However, there were some limitations in our study. Firstly, there were no further *in vivo* experiments to validate these results. In future studies, we propose the following experimental approaches to validate our findings: (i) the interaction between active ingredients and hub genes can be verified through surface plasmon resonance technology and cell thermal shift analysis. (ii) The function and mechanism of hub genes in pediatric influenza were verified through gene knockout/overexpression experiments or clinical samples. (iii) Conducting the *in vitro* antiviral experiments on BKN and its active ingredients. (iv) Animal models, i.e., develop animal models to study the therapeutic effect of BKN. Furthermore, due to ethical constraints limiting the amount of blood collected per child, conducting comprehensive mechanistic research within a restricted timeframe becomes challenging and represents a major limitation. To validate our findings effectively, it is imperative to conduct further mechanism analysis on a larger sample size.

## 5 Conclusion

In summary, based on network pharmacology, we had revealed the key components (β-sitosterol, Peimisine, and sevcoridinine), core targets (PPARG, MMP2, GSK3B, PARP1, CCNA2, and IGF1), and enriched pathways of BKN in treating pediatric influenza; Based on comprehensive bioinformatics analysis, we screened hub genes (IFI27 and OTOF) related with BKN in the progression of pediatric influenza. Molecular docking demonstrated that these hub genes and key components had excellent binding activity, and molecular dynamics simulations further confirmed the stable and compact structure of the complexes between Peimisine-IFI27 and Peimisine-OTOF. This study explores potential therapeutic drugs for pediatric influenza based on the key components and genes mediated by BKN.

## Data Availability

The datasets presented in this study can be found in online repositories. The names of the repository/repositories and accession number(s) can be found in the article/Supplementary Material.
