# Supplementary material for: Integrating multi-dimensional data to reveal the mechanisms and molecular targets of baikening granules for treatment of pediatric influenza
Source: Front Mol Biosci. 2025 Jul 11;12:1637980. doi: 10.3389/fmolb.2025.1637980 (PMC12289495; doi:10.3389/fmolb.2025.1637980)
Supplement: Supplementary file 1 [file Supplementaryfile1.docx]

Supplementary Material

# Supplementary Data

**1.1 Supplementary Table**

**Supplementary Table 1** Characteristics of pediatric influenza patients and healthy individuals without any other diseases.

| Geo accession | Age (Months) | Sex | Type |
| --- | --- | --- | --- |
| GSM844147 | 19 | Female | influenza |
| GSM844148 | 1.25 | Female | influenza |
| GSM844149 | 48 | Male | influenza |
| GSM844150 | 4 | Male | influenza |
| GSM844151 | 17 | Male | influenza |
| GSM844152 | 48 | Female | influenza |
| GSM844153 | 1.5 | Male | influenza |
| GSM844154 | 5 | Male | influenza |
| GSM844155 | 0.75 | Male | influenza |
| GSM844156 | 18 | Female | influenza |
| GSM844160 | 11 | Female | influenza |
| GSM844161 | 6 | Male | influenza |
| GSM844179 | 4 | Female | influenza |
| GSM844186 | 1.25 | Male | influenza |
| GSM844191 | 0.5 | Female | influenza |
| GSM844217 | 18 | Female | influenza |
| GSM844218 | 1.5 | Female | influenza |
| GSM844219 | 72 | Male | influenza |
| GSM844221 | 1.5 | Male | influenza |
| GSM844223 | 4.5 | Male | influenza |
| GSM844224 | 1.25 | Female | influenza |
| GSM844225 | 132 | Female | influenza |
| GSM844226 | 24 | Female | influenza |
| GSM844227 | 0.75 | Male | influenza |
| GSM844228 | 0.5 | Female | influenza |
| GSM844231 | 18 | Female | influenza |
| GSM844232 | 120 | Female | influenza |
| GSM844233 | 48 | Male | influenza |
| GSM844136 | 72 | Male | control |
| GSM844137 | 19 | Female | control |
| GSM844138 | 3 | Male | control |
| GSM844139 | 3 | Male | control |
| GSM844140 | 11 | Female | control |
| GSM844141 | 11 | Female | control |
| GSM844142 | 10 | Male | control |
| GSM844143 | 10 | Male | control |
| GSM844144 | 48 | Female | control |
| GSM844170 | 8 | Male | control |
| GSM844171 | 5 | Female | control |
| GSM844194 | 13 | Female | control |
| GSM844195 | 10 | Male | control |
| GSM844196 | 24 | Male | control |
| GSM844197 | 28 | Male | control |
| GSM844198 | 20 | Male | control |
| GSM844199 | 10 | Male | control |
| GSM844200 | 6 | Male | control |
| GSM844201 | 18 | Female | control |
| GSM844203 | 0.5 | Male | control |
| GSM844204 | 7.5 | Male | control |
| GSM844205 | 6 | Female | control |

**Supplementary Table 2** Characteristics of active ingredients in BKN.

| No | Herb | Molecule ID | Molecule name | OB (%) | | DL |
| --- | --- | --- | --- | --- | --- | --- |
| 1 | Pingbeimu | MOL010176 | [Cordiline](https://old.tcmsp-e.com/molecule.php?qn=10176) | 37.99 | 0.21 | |
| 2 | Pingbeimu | MOL010181 | Pingbeimine C | 31.88 | 0.58 | |
| 3 | Pingbeimu | MOL000359 | Sitosterol | 36.91 | 0.75 | |
| 4 | Pingbeimu | MOL004440 | Peimisine | 57.40 | 0.81 | |
| 5 | Pingbeimu | MOL009593 | Verticinone | 60.07 | 0.67 | |
| 6 | Pingbeimu | MOL010190 | Sevcoridinine | 63.34 | 0.87 | |
| 7 | Pingbeimu | MOL010193 | Ussuriendine | 69.46 | 0.43 | |
| 8 | Pingbeimu | MOL010197 | Peiminine/Verticinone | 52.43 | 0.67 | |
| 9 | Baiguoren | MOL001987 | β-sitosterol | 33.94 | 0.70 | |
| 10 | Baiguoren | MOL011586 | Ginkgolide B | 44.38 | 0.73 | |
| 11 | Baiguoren | MOL011587 | Ginkgolide C | 48.33 | 0.73 | |
| 12 | Baiguoren | MOL000519 | Coniferin | 31.11 | 0.32 | |
| 13 | Qingdai | MOL001781 | Indigo | 38.20 | 0.26 | |
| 14 | Qingdai | MOL011100 | Bisindigotin | 41.66 | 0.39 | |
| 15 | Qingdai | MOL011105 | Indican | 34.90 | 0.23 | |
| 16 | Qingdai | MOL011332 | 10h-indolo,[3,2-b],quinoline | 54.57 | 0.22 | |
| 17 | Qingdai | MOL011335 | Isoindigo | 94.30 | 0.26 | |
| 18 | Qingdai | MOL000358 | Beta-sitosterol | 36.91 | 0.75 | |
| 19 | Qingdai | MOL002322 | Isovitexin | 31.29 | 0.72 | |
| 20 | Qingdai | MOL002309 | Indirubin | 48.59 | 0.26 | |

**Supplementary Table 3** 58 Intersection genes between differentially expressed genes of pediatric influenza and BKN targets.

| No | Gene | Gene | Gene |
| --- | --- | --- | --- |
| 1 | CCNA2 | DHFR | BST1 |
| 2 | CFB | WAS | IMPDH2 |
| 3 | SELP | CTSD | RNASE3 |
| 4 | MAOB | PNP | IGF1R |
| 5 | CHEK1 | PPARG | B3GAT1 |
| 6 | FGFR1 | TGFBR1 | CYP19A1 |
| 7 | MMP8 | LCK | GSTA1 |
| 8 | PDE4D | NR3C2 | MMP2 |
| 9 | AKR1B1 | HSD17B1 | BLVRB |
| 10 | GSK3B | PARP1 | PPARA |
| 11 | PDE3B | SYK | IGF1 |
| 12 | CASP7 | PIK3CG | ATOX1 |
| 13 | KIF11 | NR1I3 | CHIT1 |
| 14 | DAPK1 | FABP5 | GNPDA1 |
| 15 | AKR1C3 | ELANE | RARA |
| 16 | EPHX2 | AHCY | NEU2 |
| 17 | CDA | MTAP | DHPS |
| 18 | PLAU | TYMP | LCN2 |
| 19 | TYMS | CRAT |  |
| 20 | CTSG | RHEB |  |

**Supplementary Table 6** 29 BKN core genes.

| No | Gene | Degree |
| --- | --- | --- |
| 1 | PPARG | 16 |
| 2 | MMP2 | 16 |
| 3 | GSK3B | 15 |
| 4 | IGF1 | 13 |
| 5 | PARP1 | 12 |
| 6 | DHFR | 10 |
| 7 | IGF1R | 10 |
| 8 | CTSG | 10 |
| 9 | TYMS | 9 |
| 10 | CCNA2 | 9 |
| 11 | CHEK1 | 8 |
| 12 | PIK3CG | 8 |
| 13 | ELANE | 8 |
| 14 | PNP | 7 |
| 15 | IMPDH2 | 7 |
| 16 | AKR1B1 | 7 |
| 17 | PPARA | 7 |
| 18 | RARA | 7 |
| 19 | CYP19A1 | 6 |
| 20 | LCN2 | 6 |
| 21 | EPHX2 | 6 |
| 22 | AHCY | 5 |
| 23 | MTAP | 5 |
| 24 | RHEB | 5 |
| 25 | TYMP | 5 |
| 26 | PLAU | 5 |
| 27 | SELP | 5 |
| 28 | MMP8 | 5 |
| 29 | SYK | 5 |
